# Lifecycle regulation of private equity in healthcare: a transatlantic governance framework

**DOI:** 10.3389/frhs.2026.1730095

**Published:** 2026-06-12

**Authors:** Hubertus J. M. Vrijhoef, Iris W. A. Boot

**Affiliations:** 1 Panaxea B.V., Den Bosch, Netherlands; 2 Department of Epidemiology, Universiteit Maastricht, Maastricht, Netherlands

**Keywords:** framework, governance, lifecycle regulation, private equity, transatlantic, healthcare

## Abstract

Private equity (PE) investment in healthcare has expanded rapidly across the United States (US) and the European Union (EU), reshaping ownership structures in primary care, specialty services, and ancillary providers. This trend has generated both optimism about capital infusion and operational efficiencies and concern about financialization, short investment horizons, and risks to care quality and equity. In this Perspective, we argue that PE participation in healthcare constitutes a systemic governance risk requiring lifecycle regulation —from acquisition through ownership to exit— rather than regulation focused narrowly on ownership status. Building on current empirical evidence and policy debates, we advance a transatlantic governance framework with five core domains: transparency, financial safeguards, clinical autonomy, quality assurance, and workforce protection. We propose context-sensitive implementation tailored to US and EU institutional realities and discuss trade-offs between regulation and investment incentives, including gaps in ownership transitions and exit phases. Finally, we outline priority research and policy experimentation to support proportionate oversight balancing innovation with patient interests and public accountability.

## Introduction

Private equity (PE) has become an increasingly visible force in healthcare delivery. Over the past decade, PE—backed acquisitions have expanded across physician practices, outpatient centres, nursing homes, and specialty care (1, 2). These investments are often presented as mechanisms for modernization and consolidation in fragmented markets (3). At the same time, critics argue that financial engineering, debt loading, and short ownership horizons may conflict with the long—term stewardship and professional autonomy expected of healthcare providers (4–6).

This debate has intensified on both sides of the Atlantic. In the US, concerns focus on rising prices, staffing reductions, and quality variation in PE—owned facilities (1). In the EU, similar questions are emerging as investor-owned chains expand within publicly financed systems traditionally organized around professional or nonprofit ownership models (2). Across these settings, PE participation increasingly raises questions not only about individual transactions but about the cumulative governance risks posed by leveraged, time-limited ownership of essential health services.

In this Perspective, we argue that PE ownership constitutes a systemic governance risk requiring oversight that follows the full investment lifecycle: from acquisition through ownership to exit. Rather than regulating ownership forms in isolation, we propose a framework organized around five domains—transparency, financial safeguards, clinical autonomy, quality assurance, and workforce protection—that can be calibrated to different sectors, institutional contexts, and investment phases. Drawing on selected empirical studies, regulatory reports, and policy debates, we situate PE within broader processes of financialization and corporate governance challenges, offering actionable guidance grounded in US and EU institutional realities for balancing investment incentives with public accountability.

### Key features, opportunities, and risks of private equity ownership

PE investment in healthcare is characterized by a distinct ownership and financing model that differs from traditional professional or nonprofit governance. PE firms typically acquire provider organisations using leveraged buyouts, consolidate fragmented markets, introduce centralized management structures, and operate within relatively short investment horizons of approximately three to seven years. These strategies are frequently accompanied by financial engineering practices, including dividend recapitalizations, sale–leasebacks, or asset restructuring, designed to optimize returns to investors (2, 4–54).

The emerging empirical literature suggests that this model can generate both opportunities and risks, with effects that vary across sectors and institutional contexts.

On the opportunity side, the evidence suggests that PE can enhance the operational performance of healthcare organizations by supplying capital and managerial expertise that enable providers to expand into new sites of care, invest in digital infrastructure, and professionalize administrative functions, particularly in fragmented outpatient and specialty markets where independent practices often face constraints in financing, management capacity, and succession planning [e.g. (8, 28, 38)]. By consolidating physician practices and introducing standardized operating procedures, PE ownership may strengthen administrative capacity and facilitate the implementation of scalable management systems (27, 45). Physicians interviewed in qualitative studies report that the transfer of administrative and bureaucratic responsibilities to specialized management teams can reduce non-clinical workload and, in some cases, improve work–life balance and career flexibility (15). PE-backed organizations may also redirect resources toward high-demand service lines and operational innovation, which can improve access and organizational responsiveness when aligned with population health needs (38, 43). At the same time, ethical and policy analyses emphasize that these potential benefits are highly context dependent and are most likely to be realized when governance arrangements preserve clinical autonomy and ensure that efficiency gains are reinvested in patient care rather than captured primarily as financial returns (8, 52).

The evidence suggests that private equity (PE) ownership can compromise the quality, accessibility, and affordability of healthcare because leveraged acquisitions and short investment horizons create strong incentives to increase returns rapidly through cost containment, service-line optimization, and market consolidation. Systematic and policy reviews conclude that these mechanisms are associated with higher healthcare spending, increased prices, and a greater likelihood that financial priorities will influence operational and clinical decisions (4–6). In nursing homes, hospitals, and physician practices, PE ownership has been linked to staffing reductions, changes in staffing expenditures, and the substitution of lower-cost labor, which may weaken organizational capacity to maintain safe and consistent care (15, 31, 45). Evidence further suggests that PE-owned organizations may shift their service mix toward more profitable procedures and become more responsive to service-line profitability, potentially reducing access to less lucrative but clinically important services (26–29). These operational changes are associated with increased utilization and spending growth in physician practices and hospitals (25, 27, 45). In some settings, these financial and operational pressures have been associated with worse patient outcomes, including higher rates of hospital adverse events, increased postoperative mortality, and poorer surgical outcomes after common inpatient procedures, lung resection, and esophagectomy (31, 42, 47, 48). PE investment also appears to accelerate consolidation in cardiovascular care, behavioral health, and hospital markets, increasing market power and potentially limiting payer leverage and patient choice (24, 39–41).

Although the magnitude and direction of these effects vary across sectors and regulatory contexts, the evidence consistently indicates that debt-driven return expectations and short investment horizons can conflict with the long-term stewardship required to sustain high-quality, equitable healthcare delivery, including in market-oriented systems such as Sweden where increased private-sector participation has been associated with concerns about inequities in access (18).

**Figure 1:**
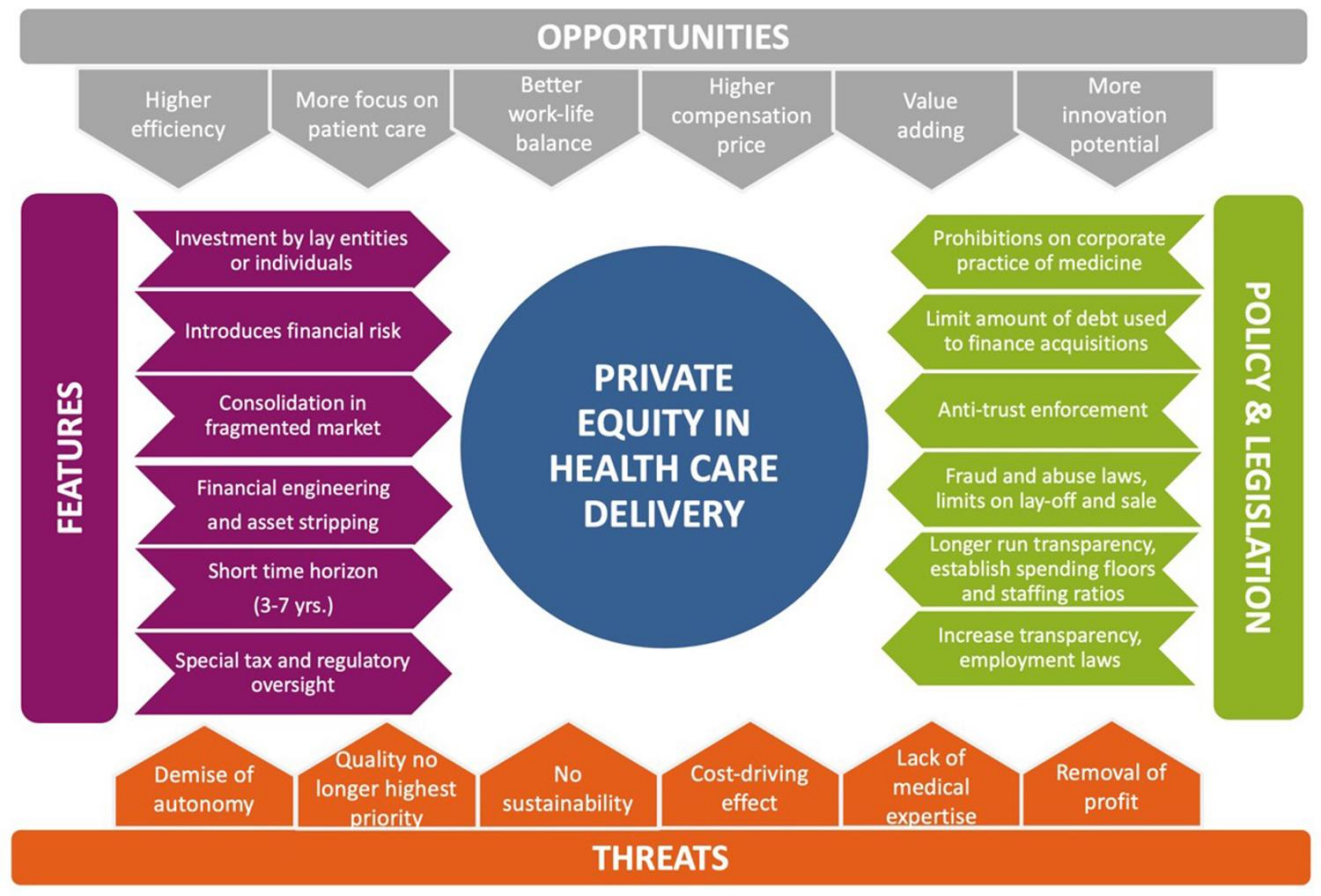
Conceptual overview of private equity in healthcare delivery

Taken together, the evidence indicates that PE ownership is neither uniformly beneficial nor uniformly harmful. Its impact depends on factors such as leverage levels, local market structure, the essential nature of services, and the strength of existing governance and regulatory safeguards. In capital-constrained environments with clear quality and access deficits, PE investment may help modernize infrastructure and expand capacity, particularly when combined with robust clinical governance. By contrast, in already consolidated or highly leveraged markets, the same financial tools can amplify risks to quality, equity, and financial resilience. This heterogeneity underscores the need for targeted, lifecycle-oriented oversight rather than blanket prohibition. Figure 1 summarizes the key ownership features, mechanisms, opportunities, risks, and policy levers described across the literature and provides a conceptual overview that informs the Perspective developed in this article.

### Institutional context matters: comparing the US and the EU

Regulatory approaches must reflect institutional realities, and structural differences between health systems justify differentiated policy instruments rather than uniform solutions. PE ownership therefore interacts differently with existing governance arrangements in the US and EU, shaping both risks and regulatory options.

In the US, fragmented financing and the central role of federal payers create opportunities to use reimbursement policy, fraud enforcement, and ownership transparency as levers for accountability. Agencies such as the Centers for Medicare & Medicaid Services, the Department of Justice, and the Federal Trade Commission already possess tools to influence provider behaviour, including conditions of participation, enforcement against abusive billing practices, and scrutiny of anticompetitive consolidation (4–8, 29, 32, 35). These instruments can be adapted to respond to PE-specific risks, for example by linking payment rules and reporting obligations to ownership structures and leverage levels.

By contrast, the EU operates through a combination of member-state health system autonomy and EU-level market and financial regulation. Ownership rules, reimbursement models, and professional governance vary widely between countries such as Germany, the Netherlands, and the Nordics, leading to different entry routes and incentives for PE investors (4–9). EU-wide reforms must often rely on financial transparency requirements, prudential regulation of investors, and competition law, while clinical governance and detailed provider oversight remain largely national. This multi-level configuration means that lifecycle-oriented oversight of PE will often require coordination between financial regulators, competition authorities, and health ministries at both EU and member-state level.

## Conceptual underpinning of oversight domains

The five oversight domains we propose are grounded in several complementary conceptual perspectives. Financialization scholarship highlights how the growing role of leveraged investors and complex capital structures can reshape organisational priorities, elevating financial returns over professional or public service logics (55). Corporate governance and principal–agent theories underscore how dispersed ownership, opaque ownership chains, and information asymmetries can weaken accountability, especially when patients and frontline professionals have limited visibility into decision-making (56). Institutional approaches to regulation emphasize that oversight must align with existing legal mandates, professional norms, and administrative capacities in different health systems (7).

Taken together, these perspectives clarify why our framework centres on transparency (addressing information asymmetries), financial safeguards (limiting excessive risk-taking), clinical autonomy and governance (protecting professional agency), quality assurance (focusing oversight on outcomes rather than ownership *per se*), and workforce protection (mitigating cost-cutting pressures that fall on staff). They also support a lifecycle lens: governance failures may emerge at acquisition, during ownership, or at exit, requiring oversight that follows the full investment trajectory rather than only the static fact of PE ownership.

### A framework for responsible oversight

Building on this conceptual and institutional analysis, we propose five interrelated domains where oversight is most likely to mitigate risks while preserving beneficial PE investment in healthcare. These domains are broadly applicable across settings but should be tailored to sector-specific conditions and the stage of the investment lifecycle.

#### Transparency

Clear visibility of ownership structures and financial flows is foundational. Without it, regulators, payers, professionals, and patients cannot assess conflicts of interest, market concentration, or exposure to financial risk. Public ownership registries, disclosure of ultimate beneficial owners, reporting of related-party transactions, and flagging of PE affiliations in provider databases can improve accountability without directly restricting investment. Enhanced transparency can also support empirical evaluation of PE impacts and inform proportionate use of other regulatory tools.

#### Financial safeguards

Mechanisms that limit excessive leverage or risky financial engineering can reduce pressure to extract short-term returns that may undermine quality or continuity of care. Options include debt caps tied to revenue stability or asset values, scrutiny of dividend recapitalizations and sale–leaseback arrangements, and closer monitoring of transactions that transfer real estate or other key assets out of operating entities. Such safeguards are not intended to deter all capital but to align financial practices with the long-term viability of essential health services and to reduce the risk of destabilizing failures.

#### Clinical autonomy and governance

Protecting professional decision-making is essential where financial incentives could distort clinical choices. Requirements that separate clinical leadership from purely financial control, establish robust clinical governance structures, and strengthen fiduciary duties to patients can reduce incentives for inappropriate service manipulation or overuse of profitable but low-value interventions. In some contexts, provisions for clinician representation in governance bodies may help counterbalance short-term financial pressures.

#### Quality assurance

Routine monitoring of outcomes, staffing levels, and patient experience is particularly important in rapidly consolidating markets and highly leveraged settings. Linking reimbursement, licensing, or participation in public programmes to validated quality indicators and minimum standards can create ownership-neutral accountability: providers are held to the same expectations regardless of whether they are PE-owned, nonprofit, or publicly owned. Where evidence suggests heightened risk in specific sectors, targeted quality audits or enhanced reporting may be warranted.

#### Workforce and care standards

Staffing reductions are a commonly reported mechanism for margin improvement under PE ownership. Minimum staffing standards, labour protections, and workforce reporting requirements can help prevent quality erosion while preserving some operational flexibility. In labour-intensive sectors such as long-term care, hospice, and behavioural health, such safeguards may be particularly critical to ensure that cost containment does not primarily occur through understaffing or precarious employment arrangements.

To support implementation, these domains can be mapped to responsible actors (e.g., health ministries, payers, financial supervisors, professional regulators), specific instruments, and monitoring indicators, recognising that the precise configuration will differ between the US and EU and across provider sectors. Table 1 provides decision guidance for policymakers, mapping context indicators to appropriate oversight intensity, tools, and responsible actors.

**Table 1:** Context-sensitive oversight framework

Context indicators	Primary risk	Oversight intensity	Key tools	Actors
High leverage (>50% debt)	Financial fragility	High	Debt caps, dividend scrutiny	Financial regulators, payers
Essential services (nursing homes, emergency room)	Quality/ access erosion	High	Staffing standards, quality metrics	Health ministries, licensing
Underserved/ rural areas	Investment deterrence	Low-moderate	Transparency focus	Competition authorities
Capital-constrained markets	Undermining benefits	Low	Light governance checks	All regulators (light touch)
Ownership transitions/ exits	Continuity disruption	High	Transaction disclosure, continuity plans	Competition and health authorities

### Trade-offs and contextual nuance

Stronger oversight is not without costs. Stricter regulation may reduce certain types of capital inflows, slow facility expansion, or discourage high-risk innovation. In underserved areas or capital-constrained sectors, limiting investor participation or imposing heavy compliance burdens could inadvertently reduce access or delay needed infrastructure upgrades (7, 8). At the same time, under-regulation can allow quality erosion, inequitable access, or financial fragility to accumulate, with significant downstream costs for patients and health systems.

For these reasons, reforms should be proportionate and context sensitive. Transparency measures typically impose relatively modest burdens and are broadly justified as a baseline. More restrictive tools, such as ownership limits, leverage caps, or stringent dividend restrictions, may be most appropriate in essential or publicly funded services where continuity of care and financial resilience are critical and where exit failures would impose large social costs. Conversely, lighter-touch approaches may suffice in less essential, more competitive markets with lower leverage and clearer evidence of net benefits from PE-driven investment. Explicitly weighing harms from over-regulation against harms from under-regulation, and periodically reviewing regulatory impacts, can help align policy intensity with demonstrated risk and adapt rules as evidence accumulates.

### Ownership transitions and exit phases

A frequently overlooked risk arises during ownership transitions, including both entry and exit phases of PE investments (6, 7). The period preceding sale may incentivize cost cutting, asset stripping, or deferred investment to maximize valuation, potentially destabilizing care delivery or degrading quality. Rapid turnover between successive PE owners or between PE and other corporate owners can also disrupt organisational continuity, undermine long-term planning, and create uncertainty for staff and patients.

Policies that require timely disclosure of pending transactions, transitional oversight for ownership changes involving essential services, and credible continuity plans can reduce these risks. Regulators may, for example, require acquirers to demonstrate financial and operational capacity to maintain services, or impose conditions on sales where continuity is at stake. Future regulation should pay particular attention to these exit dynamics and their distributional effects, which remain underexamined in both research and policy debates but are central to a lifecycle-oriented oversight approach.

### Future directions for research and policy

Empirical evidence on PE ownership remains uneven across sectors and geographies, particularly in the EU (2). Future work should move beyond descriptive mapping of ownership patterns toward causal evaluations of how ownership structures, financial practices, and specific regulatory tools influence outcomes such as quality, access, equity, and financial resilience. Natural experiments, difference-in-differences analyses, and cross-country comparisons exploiting variation in regulatory regimes could provide stronger inference about mechanisms and trade-offs.

For policymakers, priority questions include: which combinations of oversight tools are most effective in high-risk sectors; how leverage thresholds and dividend restrictions affect both financial stability and investment incentives; and how transparency and reporting rules can best support accountability without imposing disproportionate administrative burdens. Pilot programmes that test graduated oversight mechanisms—such as tiered safeguards based on leverage, service criticality, or market concentration—may enable learning without premature over- or under-regulation. A clearer evidence base on these questions is essential for designing proportionate, lifecycle-oriented regulation that balances innovation and investment with the protection of patient interests and public accountability.

## Discussion

PE's growing role in healthcare presents neither a simple threat nor an unqualified opportunity. It reflects broader financialization trends that can bring both capital and managerial expertise, but also heightened leverage, shorter investment horizons, and new forms of governance risk. The central policy challenge is therefore not whether PE should participate in healthcare, but under what conditions such participation can be made compatible with patient welfare, professional integrity, and long-term system sustainability.

In this Perspective, we have argued that PE ownership in healthcare should be treated as a systemic governance risk that warrants lifecycle-oriented oversight, following investments from acquisition through ownership to exit. Rather than focusing solely on ownership form, we propose a framework organised around five domains—transparency, financial safeguards, clinical autonomy and governance, quality assurance, and workforce protection—that can be adapted to different sectors and institutional configurations. In the US and EU, these domains will necessarily be implemented through distinct regulatory strategies, reflecting differences in financing arrangements, legal mandates, and administrative capacity, but they share a common aim: ensuring that financial innovation serves, rather than undermines, the core mission of healthcare.

Recognising trade-offs is central to designing proportionate and context-sensitive oversight. Stricter safeguards may reduce some types of capital inflows or slow expansion in certain settings, yet under-regulation can allow quality erosion, inequities, and financial fragility to accumulate. By reframing the debate from ownership ideology to risk management and accountability, policymakers can calibrate oversight intensity to demonstrable risks, paying particular attention to highly leveraged investments, essential services, and ownership transitions. Continued empirical research —particularly causal evaluations of regulatory tools and lifecycle dynamics— alongside structured policy experimentation will be essential to refine these approaches and to build an evidence base for sustainable, accountable engagement of PE in healthcare.

## Data Availability

The original contributions presented in the study are included in the article, further inquiries can be directed to the corresponding author.

## References

[1] Blumenthal D. Private Equity's Role in Health Care (explainer). New York: Commonwealth Fund (2023).

[2] Rechel B Tille F Groenewegen P Timans R Fattore G Rohrer-Herold K. Private equity investment in Europe's primary care sector—a call for research and policy action. Eur J Public Health. (2023) 33(3):354–5. 10.1093/eurpub/ckad061 37053379 PMC10234640

[3] Bervell J Song Z. How Private Equity Deals are Reshaping Your Health Care. New York: Commonwealth Fund (2025).

[4] Cai C Song Z. A policy framework for the growing influence of private equity in health care delivery. JAMA. (2023) 329(18):1545–6. 10.1001/jama.2023.2801 37052901 PMC10699936

[5] Borsa A Bejarano G Ellen M Bruch JD. Evaluating trends in private equity ownership and impacts on health outcomes, costs, and quality: a systematic review. Br Med J. (2023) 382:e075244. 10.1136/bmj-2023-075244 37468157 PMC10354830

[6] Fuse Brown E Hall M. Private equity and the corporatization of health care. Stanford Law Rev. (2024) 76:527–96. Available online at: https://review.law.stanford.edu/wp-content/uploads/sites/3/2024/03/Fuse-Brown-Hall-76-Stan.-L.-Rev.-527.pdf (Accessed October 21, 2025).

[7] Tracey M Schulmann K Tille K Rice T Mercille J Timans R. What are the policy options for regulation private equity involvement in health care? A review of policies implemented or considered in seven high-income countries. Health Policy. (2025) 156:105312. 10.1016/j.healthpol.2025.105312 40250333

[8] Unruh L Rice T. Private equity expansion and impacts in United States healthcare. Health Policy. (2025) 155:105266. 10.1016/j.healthpol.2025.105266 40036910

[9] Burstrom B Burstrom K Nilsson G Tomson G Whitehead M Winblad U. Equity aspects of the primary health care choice reform in Sweden—a scoping review. Int J Equity Health. (2017) 16(1):29. 10.1186/s12939-017-0524-z 28129771 PMC5273847

[10] Kullberg L Blomqvist P Winblad U. Market-orienting reforms in rural health care in Sweden: how can equity in access be preserved? Int J Equity Health. (2018) 17(1):123. 10.1186/s12939-018-0819-8 30119665 PMC6098624

[11] Burstrom B. What is happening in Sweden? Int J Health Serv. (2019) 49(2):204–11. 10.1177/0020731418822236 30638431

[12] Bos A Kruse FM Jeurissen PPT. For-profit nursing homes in The Netherlands: what factors explain their rise? Int J Health Serv. (2020) 50(4):431–43. 10.1177/0020731420915658 32276563 PMC7441333

[13] Mosquera PA San Sebastian M Burstrom B Hurtig AK Gustafsson PE. Performing through privatization: an ecological natural experiment of the impact of the Swedish free choice reform on ambulatory care sensitive conditions. Front Public Health. (2021) 9:504998. 10.3389/fpubh.2021.504998 34136446 PMC8200664

[14] Frederiksson M Isaksson D. Fifteen years with patient choice and free establishment in Swedish primary healthcare: what do we know? Scan J Public Health. (2022) 50(7):852–63. 10.1177/14034948221095365 PMC9578085 35596549

[15] Nolte TN Miedaner F Sulz S. Physicians' perspectives regarding private equity transaction in outpatient health care—a scoping review and qualitative analysis. Int J Environ Res Publ Health. (2022) 19:15480. 10.3390/ijerph192315480 PMC9737937 36497553

[16] Winkelmann J Rossi JG Van Ginneken E. Oral health care in Europe: financing, access and provision. Health Syst Transit. (2022) 24(2):1–176. Available online at: https://iris.who.int/server/api/core/bitstreams/cc278e23-0894-4117-a51d-28b1539f2174/content (Accessed October 21, 2025). 35833482

[17] Glenngard AH. Exploring differences between public and private providers in primary care: findings from a large Swedish region. Health Econ Policy Law. (2023) 18(3):219–33. 10.1017/S1744133122000251 36349928

[18] Gustafsson PE Fonseca-Rodiguez O San Sebastian M Burstrom B Mosquera PA. Evaluating the impact of the 2010 Swedish choice in primary health care on avoidable hospitalization and socioeconomic inequities: an interrupted time series analysis using register data. BMC Health Serv Res. (2024) 24(1):972. 10.1186/s12913-024-11434-w 39174988 PMC11342640

[19] Braun RT Yun H Casalino LP Myslinksi Z Kuwonza FM Jung HY. Comparative performance of private equity-owned US nursing homes during the COVID-19 pandemic. JAMA Netw Open. (2020) 3(10):e2026702. 10.1001/jamanetworkopen.2020.26702 33112402 PMC7593807

[20] Tanne JH. US Congress investigates effects of $80bn private equity industry on government healthcare programme. Br Med J. (2020) 370:m3490. 10.1136/bmj.m3490 32895222

[21] Braun RT Jung HY Casalino LP Myslinski Z Unruh MA. Association of private equity investment in US nursing homes with the quality and cost of care for long-stay residents. JAMA Health Forum. (2021) 2(11):e213817. 10.1001/jamahealthforum.2021.3817 35977267 PMC8796926

[22] Geyman J. Investor-owned health care: the hidden blight om America's “system”. Int J Health Serv. (2021) 51(4):494–500. 10.1177/00207314211015806 33988483

[23] Creadore A Desai S Li SJ Lee KJ Bui ATN Villa-Ruiz C. Insurance acceptance, appointment wait time, and dermatologist access across practice types in the US. JAMA Dermatol. (2021) 157(2):181–8. 10.1001/jamadermatol.2020.5173 33439219 PMC7807384

[24] Neely MT Carmichael D. Profiting on crisis: how predatory financial investors have worsened inequality in the coronavirus crisis. Am Behav Sci. (2021) 65(12):1649–70. 10.1177/00027642211003162 38603051 PMC7992100

[25] Offodile AC Cerullo M Bindal M Rauh-Hain JA Ho V. Private equity investments in health care: an overview of hospital and health system leveraged buyouts, 2003–17. Health Aff. (2021) 40(5):719–26. 10.1377/hlthaff.2020.01535 33939504

[26] Cerullo M Yang KK Roberts J McDevitt RC Offodile AC. Private equity acquisition and responsiveness to service-line profitability at short-term acute care hospitals. Health Aff. (2021) 40(11):1697–705. 10.1377/hlthaff.2021.00541 34724425

[27] Singh Y Song Z Polsky D Bruch JD Zhu JM. Association of private equity acquisition of physician practices with changes in health care spending and utilization. JAMA Health Forum. (2022) 3(9):e222886. 10.1001/jamahealthforum.2022.2886 36218927 PMC9440392

[28] Singh Y Zhu JM Polsky D Song Z. Geographic variation in private equity penetration across select office-based physician specialities in the US. JAMA Health Forum. (2022) 3(4):e220825. 10.1001/jamahealthforum.2022.0825 35977319 PMC9055458

[29] Crosson FJ Ostrer IR Gross CP. Private equity in US health care—now cradle to grave? JAMA Intern Med. (2023) 183(6):511–2. 10.1001/jamainternmed.2023.0324 37126342

[30] Klein DJ Veillard J Brown A. Aligning goals of for-profit, and not-for-profit, and public healthcare organizations by governing for quality: a model for change in the U.S. and other jurisdictions. Healthcare Manag Forum. (2023) 36(4):246–8. 10.1177/08404704231160274 PMC10291483 36959688

[31] Kannan S Bruch JD Song Z. Changes in hospital adverse events and patient outcomes associated with private equity acquisition. JAMA. (2023) 330(24):2365–75. 10.1001/jama.2023.23147 38147093 PMC10751598

[32] Braun RJ Unruh MA Stevenson DG Prigerson HG Fernandez R Yao LZ. Changes in diagnoses and site of care for patients receiving hospice care from agencies acquired by private equity firms and publicly traded companies. JAMA Netw Open. (2023) 6(9):e2334582. 10.1001/jamanetworkopen.2023.34582 37747735 PMC10520742

[33] Aldridge MD Hunt LJ Halloran Z Harrison KL. Private equity acquisitions of hospices are increasing; ownership remains opaque. Health Aff. (2024) 43(9):1306–10. 10.1377/hlthaff.2023.01671 PMC12674456 39226494

[34] Bejarano G Eubanks JE Braun RT. Trends in private equity acquisition of pain management practices. JAMA Netw Open. (2024) 7(12):e2451688. 10.1001/jamanetworkopen.2024.51688 39699898 PMC11659914

[35] Williams D Jr Fernandez R Stevenson D Unruh M Braun RT. Nursing home finances associated with real estate investment trust and private equity investments. Health Aff Sch. (2024) 2(4):qxae037. 10.1093/haschl/qxae037 38756179 PMC11034530

[36] Chen AC Skinner RJ Braun RT Konetzka RT Stevenson DG Grabowski DC. New CMS nursing home ownership data: major gaps and discrepancies. Health Aff. (2024) 43(3):318–26. 10.1377/hlthaff.2023.01110 38437601

[37] Saulsberry L. Medicaid privatization: has the invisible hand led us astray? JCO Oncol Pract. (2024) 20(5):599–601. 10.1200/OP.23.00820 38364195 PMC11163970

[38] Cai C Song Z. Protecting patients and society in an era of private equity provider ownership: challenges and opportunities for policy. Health Aff. (2024) 43(5):666–73. 10.1377/hlthaff.2023.00942 PMC11745941 38709967

[39] Singh Y Reddy M Whaley C. Trends in private equity consolidation in cardiovascular care. JAMA Health Forum. (2024) 5(6):e241478. 10.1001/jamahealthforum.2024.1478 38874961 PMC11179124

[40] Richards MR Whaley CM. Hospital behavior over the private equity life cycle. J Health Econ. (2024) 97:102902. 10.1016/j.healeco.2024.102902 38861907 PMC11392649

[41] Zhu JM Greenberg E King M Busch S. Geographic penetration of private equity ownership in outpatient and residential behavioral health. JAMA Psychiatry. (2024) 81(7):732–5. 10.1001/jamapsychiatry.2024.0825 38691384 PMC11063916

[42] Diaz A Mead M Rohde S Kunnath N Dimick JB Ibrahim AM. Hospitals acquired by private equity firms: increased postoperative mortality for common inpatient surgeries. Health Aff. (2025) 44(5):554–62. 10.1377/hlthaff.2024.01102 40324137

[43] Singh Y Cardenas GB Torabzadeh H Whaley CM Borkar D. Private equity-owned physician practices decreased access to retinal detachment surgery, 2014–22. Health Aff. (2025) 44(5):589–96. 10.1377/hlthaff.2024.01204 40267368

[44] Smith K. Common institutional ownership and the erosion of competition in the America health insurance market: a quantitative analysis. Health Policy. (2025) 156:105316. 10.1016/j.healthpol.2025.105316 40228424

[45] Kannan S Song Z. Variation in hospital salary expenditures and utilization changes after private equity acquisition, 2005–19. Health Aff. (2025) 44(2):206–14. 10.1377/hlthaff.2024.00687 PMC12455575 39899773

[46] Rahman MI. Understanding private equity-owned HHAs in the US: a performance comparison between pe-owned and non-pe-owned agencies. Health Policy. (2025) 153:105250. 10.1016/j.healthpol.2025.105250 39847920

[47] Williams JE Schaefer SL Jacobs RC Jacobson-Davies F Ibrahim AM Odell DD. Postoperative outcomes following lung resection performed at private equity-acquired hospitals. J Thorac Cardiovasc Surg. (2025) 169(6):1585–92. 10.1016/j.jtcvs.2024.12.032 39824345

[48] Williams JE Schaefer SL Jacobs RC Ibrahim AM Odell DD. Esophagectomy trends and postoperative outcomes at private equity-acquired health centers. JAMA Surg. (2025) 160(3):296–302. 10.1001/jamasurg.2024.5920 39745696 PMC11904734

[49] Harrington CA. Wealth extraction from a nursing home chain with individual, private equity, and real estate owners. Int J Soc Determinants Health Health Serv. (2025) 55(4):441–54. 10.1177/27551938251335565 40350782 PMC12371139

[50] Connolly JE Guido M Girard A Braun RT Emanuel EJ. Changes in payer mix associated with private equity acquisition of ophthalmology practices. JAMA Netw Open. (2025) 8(5):e2512629. 10.1001/jamanetworkopen.2025.12629 40434776 PMC12120645

[51] Shields MC Yang Y Busch SH. Private equity among US psychiatric hospitals. JAMA Psychiatry. (2025) 82:e250689. 10.1001/jamapsychiatry.2025.0689 PMC12096323 40397464

[52] Statchen T Grogan CM. Health inequity profiteering by private equity firms. AMA J Ethics. (2025) 27(5):E361–8. 10.1001/amajethics.2025.361 40315111

[53] Ramesh T Blumenthal D Tsai TC. Hospitals as tenants: the rise of real estate investment trusts in health-care delivery. Lancet. (2025) 407(25):657–9. 10.1016/S0140-6736(25)00498-2 40179930

[54] Zhu DT Song Z Kannan S Cai CL Bajaj SS Gondi S. Private equity ownership of US opioid treatment programs. JAMA Psychiatry. (2025) 82(2):204–6. 10.1001/jamapsychiatry.2024.4011 39661342 PMC11800012

[55] Hunter BM Chakravarthi I Marathe S Murray SR. Financialisation and the reshaping of private equity: a case study in India. Social Health Illn. (2025) 47(4):e70041. 10.1111/1467-9566.70041 PMC12082753 40377098

[56] Millson R Ward M. Corporate governance criteria as applied in private equity investments. S Afr J Bus Manage. (2025) 36(1):73085. 10.4102/sajbm.v36i1.622

